# Status of Registered Clinical Regulatory Trials of Non-communicable Diseases in the Pre- and Post-COVID-19 Era: A Web-Based Study

**DOI:** 10.7759/cureus.117881

**Published:** 2026-10-13

**Authors:** Raakhi K Tripathi, Urjita R Kapadia, Avanti V Kajrolkar, Urwashi I Parmar

**Affiliations:** 1 Pharmacology &amp; Therapeutics, Seth GS Medical College and King Edward Memorial Hospital, Mumbai, IND

**Keywords:** india, industry-sponsored research, patient recruitment, research design, united states

## Abstract

Introduction: Non-communicable diseases (NCDs) are a major global health burden and constitute the majority of all registered clinical trials. Clinical trials are vital for developing effective management strategies for NCDs. The COVID-19 pandemic significantly disrupted the conduct of clinical trials worldwide. This study aimed to compare the status of registered clinical regulatory trials for major NCDs in India and the USA before and after the pandemic.

Methodology: A retrospective, web-based analytical study was conducted. Data were collected from CTRI (Clinical Trials Registry - India) and ClinicalTrials.gov (USA) for the following NCDs: diabetes mellitus, breast cancer, asthma, hypertension, chronic obstructive pulmonary disease (COPD), ischemic heart disease, and stroke across the pre-COVID (January 2018-January 2020) and post-COVID (June 2023-June 2025) periods. Pharmaceutical industry- sponsored, interventional drug trials with regulatory approval were included. Categorical variables were analyzed by the chi-square test and Fisher's exact test with odds ratios and 95% confidence intervals. Continuous variables were analyzed using the Mann-Whitney U test. The Benjamini-Hochberg procedure was applied to control the false discovery rate for multiple comparisons, and an adjusted p-value of <0.05 was considered statistically significant.

Results: Total number of trials increased from 456 to 483 in the post-COVID period, with trials in India increasing from 95 to 141, while in the USA, they decreased slightly from 361 to 342. In India, trials for COPD increased significantly (p-value = 0.035), while trials for diabetes decreased significantly (p-value = 0.0063) in the post-COVID period. A significant decrease was seen in Phase 4 trials in the USA in the post-COVID period (p-value = 0.035). In the post-COVID period, in both countries, a significantly larger number of trials were open to recruitment and significantly fewer trials had recruitment closed. In the USA, there was a significant decrease in the number of terminated trials and trials with an unknown status. Additionally, trials in India had a significantly lower median number of study sites in the post-COVID period.

Conclusion: Overall NCD trial numbers have largely recovered post-COVID. However, certain elements were impacted; for example, in India, trials for COPD showed a significant rise, while trials for diabetes declined significantly. Phase 4 trials declined significantly in the USA. Changes were seen in recruitment status in both countries, and the median number of study sites decreased significantly in India.

## Introduction

Non-communicable diseases (NCDs) constitute a major public health challenge due to their high and rising prevalence and account for 81% of all the clinical trials registered between 1999 and 2025 [1]. Clinical trials are vital for the development of effective management strategies for NCDs, and interruptions in trial activity can hinder progress in their management. The COVID-19 pandemic severely disrupted the management and conduct of clinical trials [2]. As resources were directed to management of the pandemic and COVID-19 research, clinical research in many therapeutic areas, including NCDs, took a backseat [3].

The WHO declared an end to the COVID-19 global health emergency on May 5, 2023 [4]. Several studies have documented the disruption of clinical research during the COVID-19 pandemic [2,5]. However, it was important to evaluate whether clinical trial numbers and their characteristics have now returned to pre-pandemic levels.

To assess this, we conducted a web-based study to compare the number and characteristics of clinical regulatory trials for selected major NCDs in India and the USA before and after the COVID-19 pandemic. India and the USA were selected for comparison to evaluate clinical trial activity in an emerging clinical research market and a historically established setting for industry-sponsored clinical research, respectively [6]. In the context of this study, clinical regulatory trials refer to pharmaceutical industry-sponsored interventional drug clinical trials that have received approval from the relevant regulatory authority.

The primary objective was to compare the number of clinical regulatory trials from the pre-COVID-19 pandemic period to the post-COVID-19 pandemic period in India and the USA for select NCDs. The secondary objective was to compare the clinical regulatory trials registered for select NCDs in the pre- and post-COVID-19 pandemic period in India and the USA in terms of therapeutic area, trial phase, number of sites, multinational/national sites, study design, blinding, comparator type, sample size, and recruitment status.

This study was previously presented as an oral presentation at NAPTICON 2025 (4th National Conference of the National Association of Pharmacology & Therapeutics) on November 28, 2025.

## Materials and methods

A retrospective web-based analytical study was conducted at the Department of Pharmacology & Therapeutics, Seth GS Medical College and KEM Hospital. Exemption from Institutional Ethics Committee review was obtained in May 2025 (EC/OA-43/2025). Data were extracted from the Clinical Trials Registry-India (CTRI) website for the Indian clinical trial data, and we compared them with data from the USA collected from the ClinicalTrials.gov website.

Data collection was done over a period of 10 months from May 2025 to February 2026. The pre-COVID-19 period was defined as 24 months before the start of the pandemic, i.e., 30 January 2018 to 29 January 2020, and the post-COVID-19 period was defined as 24 months after the end of the pandemic, i.e., 1 June 2023 to 1 June 2025. The pre-COVID-19 and post-COVID-19 periods were each defined as 24 months (two years) to provide comparable observation windows. A two-year post-pandemic period was chosen to allow clinical research activities to recover from the immediate disruptions of the pandemic and to prevent residual or carryover effects of the pandemic. The period of the pandemic was excluded because the objective was to compare the status of trials from the pre-pandemic baseline with the subsequent post-pandemic period rather than to assess the acute disruption during the pandemic.

The inclusion criteria for Indian data collected from CTRI consisted of "interventional" studies, "regulatory clearance status from DCGI" as "Approved", and "drug" studies with the primary sponsor as a "pharmaceutical industry". For the USA data collected from ClinicalTrials.gov, the location was selected as "United States of America" and "interventional" studies funded by "industry" and involving a "US FDA-regulated drug product" were included. The NCDs included in both India and the USA were ischemic heart disease (IHD), stroke, hypertension, asthma, chronic obstructive pulmonary disease (COPD), diabetes mellitus, and breast cancer, using the same search terms. The above-listed diseases were chosen as they account for the highest burden amongst all NCDs [7]. Breast cancer was selected as it is the most prevalent cancer in India and the USA [8,9]. We excluded studies on NCDs with COVID-19.

For each trial, we recorded data on therapeutic area, trial phase, number of sites, whether the sites were national or multinational, study design, blinding, comparator type, sample size, and recruitment status. Trials were labelled as national or multinational based on the study site locations as reported in the registries. Trials with reported study sites only in India (on the CTRI website) and the USA (on the ClinicalTrials.gov website) were considered national, whereas reported locations containing any one other country were classified as multinational. Detailed search strategy and data extraction process are provided in figures and tables in the Appendix.

Categorical variables were expressed as numbers and percentages, and quantitative variables were expressed as mean + standard deviation or median and interquartile range, as appropriate. Categorical data were analysed using Pearson's chi-square test, and Fisher's exact test was used for comparisons with small cell frequencies. Normality of continuous data was assessed by the Shapiro-Wilk test. Non-normally distributed variables were analysed by the Mann-Whitney U test. Effect sizes for categorical comparisons were reported as odds ratios (ORs) with 95% confidence intervals (CIs), while rank-biserial correlation was used to report the effect size for Mann-Whitney U comparisons. The Benjamini-Hochberg (BH) procedure was used to control the false discovery rate, with correction applied separately to related groups of comparisons. BH correction was not applied to the analyses of the "number of study sites" and "sample size" as they did not involve multiple comparisons of the same outcome. A p-value of < 0.05 was considered statistically significant. Adjusted p-values were reported throughout the Results section for variables to which the BH correction was applied, while unadjusted p-values were reported for the analysis of the number of study sites and sample size.Statistical analyses were performed using GraphPad Prism (version 10.0, GraphPad Software, San Diego, CA), StatsKingdom (StatsKingdom, Statistics Kingdom, Melbourne, Australia), and jamovi (version 2.7.38; The jamovi Project, Australia).

## Results

In the pre-COVID period, the total number of regulatory clinical trials for NCDs was 456, which increased to 483 in the post-COVID period. Out of the 456 trials in the pre-COVID period, 95 were in India and 361 were in the USA, whereas out of the 483 trials in the post-COVID period, 141 were in India and 342 were in the USA.

According to the therapeutic area, the total number of clinical trials for diabetes was 132 in the pre-COVID period, which decreased to 123 in the post-COVID period. Corresponding numbers for breast cancer were 186 and 177, for asthma 48 and 64, for hypertension 23 and 32, for COPD 26 and 48, for IHD 22 and 24, and for stroke 19 and 15 in the pre-COVID and post-COVID periods, respectively. In India, there was a significant decrease in diabetes trials (p-value = 0.0063) and a significant increase in COPD trials (p-value = 0.035). Trials for breast cancer, asthma, stroke, and IHD also rose, while those for hypertension declined slightly. Contrastingly, there was an increasing trend observed in trials for hypertension in the post-COVID period in the USA; however, the change was found to be non-significant (p-value = 0.1176). Similar to India, trials for COPD and asthma in the USA showed a rise, but the changes were not found to be statistically significant, whereas IHD, breast cancer, and stroke showed a statistically non-significant decline (Table 1).

**Table 1:** Distribution of trials as per therapeutic area Data are represented as numbers and percentages. Categorical data were analysed using Pearson's chi-square test or Fisher's exact test, as appropriate. # indicates that Fisher's exact test was applied; therefore, no χ² statistic is reported. Adjusted p-values were obtained using the Benjamini-Hochberg procedure to control the false discovery rate. Odds ratios (ORs) are presented with 95% confidence intervals (CIs) and represent the odds of a trial belonging to the given category in the pre-COVID period relative to the post-COVID period. * represents adjusted p-value < 0.05.

Therapeutic area	India						USA					
	Pre-COVID period n=95	Post-COVID period n=141	χ²	P-value	Adjusted p-value	OR (95% CI)	Pre-COVID period n=361	Post-COVID period n=342	χ²	P-value	Adjusted p-value	OR (95% CI)
Diabetes	35 (36.8%)	25 (17.73%)	10.934	0.0009	0.0063*	2.71 (1.48-4.93)	97 (26.86%)	98 (28.65%)	0.279	0.5972	0.6217	0.915 (0.657-1.27)
Breast cancer	25 (26.3%)	41 (29.07%)	0.215	0.6429	0.6429	0.871 (0.486-1.56)	161 (44.59%)	136 (39.76%)	1.681	0.1948	0.4545	1.22 (0.903-1.65)
Asthma	10 (10.52%)	24 (17.02%)	1.942	0.1635	0.2861	0.574 (0.261-1.26)	38 (10.52%)	40 (11.69%)	0.244	0.6217	0.6217	0.888 (0.555-1.42)
Hypertension	14 (14.73%)	11 (7.80%)	2.883	0.0971	0.2266	2.04 (0.884-4.72)	9 (2.49%)	21 (6.14%)	5.719	0.0168	0.1176	0.391(0.176-0.866)
COPD	5 (5.26%)	23 (16.31%)	6.626	0.01	0.035*	0.285 (0.104-0.779)	21 (5.81%)	25 (7.30%)	0.64	0.4237	0.6202	0.783 (0.430-1.43)
Ischemic Heart Disease	4 (4.21%)	11 (7.80%)	#	0.4152	0.5601	0.519 (0.160-1.68)	18 (4.98%)	13 (3.80%)	0.5850	0.4430	0.6202	1.33 (0.641-2.75)
Stroke	2 (2.10%)	6 (4.25%)	#	0.4801	0.5601	0.484 (0.0956-2.45)	17 (4.70%)	9 (2.63%)	2.128	0.1446	0.4545	1.83 (0.804-4.16)

In terms of trial phase, the number of trials in the pre-COVID period and post-COVID period, respectively, for Phase 1 trials were 122 and 114, for Phase 2 trials were 133 and 138, for Phase 3 trials were 153 and 178, for Phase 4 trials were 35 and 31, and for BA/BE studies were 13 and 22, respectively. In India, Phase 3 trials remained the most common in both periods, constituting about 59% of the trials, whereas in the USA, Phase 1 and Phase 2 trials constituted the majority across both periods. The remaining distribution of trial phases remained largely similar across the two periods. There was a significant decrease in the number of Phase 4 trials in the USA in the post-COVID period (p-value = 0.035). The distribution of the remaining trial phases showed no substantial change between the two periods (Table 2).

**Table 2:** Distribution of trials as per trial phase Data are presented as numbers and percentages. Categorical data were analysed using Pearson's chi-square test or Fisher's exact test, as appropriate. # indicates that Fisher's exact test was applied; therefore, no χ² statistic is reported. Adjusted p-values were obtained using the Benjamini-Hochberg procedure to control the false discovery rate. Odds ratios (ORs) are presented with 95% confidence intervals (CIs) and represent the odds of a trial belonging to the given category in the pre-COVID period relative to the post-COVID period. * represents an adjusted p-value < 0.05. BA/BE: bioavailability/bioequivalence

Trial phase	India						USA					
	Pre-COVID period, n=95	Post-COVID period, n=141	χ²	P-value	Adjusted P-value	OR (95% CI)	Pre-COVID period, n=361	Post-COVID period, n=342	χ²	P-value	Adjusted P-value	OR (95% CI)
Phase 1	1 (1.05%)	1 (0.71%)	#	1	1	1.49 (0.0920-24.1)	121 (33.51%)	113 (33.04%)	0.018	0.8933	1	1.02 (0.746-1.40)
Phase 2	12 (12.63%)	10 (7.09%)	2.0603	0.1512	0.756	1.89 (0.783- 4.58)	121 (33.51%)	128 (37.42%)	1.173	0.2788	0.697	0.843 (0.619-1.15)
Phase 3	57 (60.00%)	84 (59.57%)	0.0043	0.9479	1	1.02 (0.599-1.73)	96 (26.59%)	94 (27.48%)	0.071	0.790	1	0.956 (0.685-1.33)
Phase 4	13 (13.68%)	24 (17.02%)	0.4781	0.4893	0.873	0.773 (0.372-1.61)	22 (6.09%)	7 (2.04%)	7.274	0.007	0.035*	3.11 (1.31-7.37)
BA/BE	12 (12.63%)	22 (15.60%)	0.4064	0.5238	0.873	1.28 (0.60-2.73)	1 (0.27%)	0 (0.00%)	#	1	1	2.85 (0.116-70.2)

In terms of study design, the number of trials in the pre-COVID period and post-COVID period were 63 and 69 for single-arm studies, 247 and 267 for randomised parallel studies, 39 and 41 for randomised crossover studies, and 107 and 106 for "Other" study designs, respectively. In India and the USA, randomized parallel trials predominated, but their percentage slightly decreased from 70.53% to 67.38% in India. In India, randomised crossover designs showed a slight increase from 12.63% to 15.60%, while the USA showed contrasting findings with a slight decrease from 7.47% to 5.55%. The remaining distribution of study designs remained largely similar across both periods.

In terms of the comparator, the numbers of trials in the pre-COVID and post-COVID periods, respectively, were 196 and 229 for active comparators, 197 and 185 for placebo comparators, and 63 and 69 for studies with no comparator. The pattern of distribution of comparators used remained largely consistent across both periods.

Regarding blinding status, the numbers of trials in the pre-COVID and post-COVID periods, respectively, were 245 and 265 for open-label trials, 9 and 8 for single-blind studies, 73 and 91 for double-blind studies, 10 and 10 for double-blind, double-dummy studies, 37 and 31 for triple-blind studies, and 82 and 78 for quadruple-blind studies. In India, an upward trend was noted in open-label trials post-COVID; however, the change was found to be non-significant (p-value = 0.138). More complex blinding types such as double-blind double dummy and triple- and quadruple-blind designs declined. In the USA, double-blind studies showed a rise from 13.85% in the pre-COVID period to 16.66% in the post-COVID period, while the remaining types showed a marginal decrease or remained mostly constant (Table 3).

**Table 3:** Distribution of trials as per study design, comparator, and blinding Data are presented as numbers and percentages. Categorical data were analysed using Pearson's chi-square test or Fisher's exact test, as appropriate. # indicates that Fisher's exact test was applied; therefore, no χ² statistic is reported. Adjusted p-values were obtained using the Benjamini-Hochberg procedure to control the false discovery rate. Odds ratios (ORs) are presented with 95% confidence intervals (CIs) and represent the odds of a trial belonging to the given category in the pre-COVID period relative to the post-COVID period. * represents adjusted p-value <0.05. "Other" study designs included non-randomised parallel, non-randomised crossover, randomised sequential, non-randomised sequential, and randomised factorial designs. Double-blind double-dummy refers to a blinding technique in which participants in both treatment groups receive active medication and matching placebo to maintain blinding despite different dosage forms or administration schedules.

Parameters	India						USA					
	Pre-COVID period n=95	Post-COVID period n=141	χ²	P-value	Adjusted P-value	OR (95% CI)	Pre-COVID period n=361	Post-COVID period n=342	χ²	P- value	Adjusted p value	OR (95% CI)
A. Design												
Single arm	16 (16.84%)	24 (17.02%)	0.0013	0.9713	0.971	0.987 (0.493-1.98)	47 (13.01%)	45 (13.15%)	0.003	0.9566	0.9566	0.988 (0.637-1.53)
Randomised parallel	67 (70.53%)	95 (67.38%)	0.2617	0.6089	0.912	1.16 (0.659-2.04)	180 (49.86%)	172 (50.29%)	0.013	0.9091	0.9566	0.983 (0.731-1.32)
Randomised crossover	12 (12.63%)	22 (15.60%)	0.4064	0.5238	0.912	0.782 (0.367-1.67)	27 (7.47%)	19 (5.55%)	1.063	0.3026	0.9566	1.37 (0.749-2.52)
Others	-	-	-	-	-	-	107 (29.63%)	106 (30.99%)	0.153	0.6961	0.9566	0.938 (0.680-1.29)
B. Comparator												
Active comparator	61 (64.21%)	92 (65.25%)	0.0268	0.8699	0.971	0.956 (0.555-1.65)	135 (37.39%)	137 (40.05%)	0.525	0.4688	0.7032	0.894 (0.660-1.21)
Placebo	18 (18.95%)	25 (17.73%)	0.0564	0.8123	0.971	1.08 (0.555-2.12)	179 (49.58%)	160 (46.78%)	0.552	0.4576	0.7032	1.12 (0.832-1.50)
NA	16 (16.84%)	24 (17.02%)	0.0013	0.9713	0.971	0.987 (0.493-1.98)	47 (13.01%)	45 (13.15%)	0.003	0.9566	0.9566	0.988 (0.637-1.53)
C. Blinding												
Open label	43 (45.26%)	85 (60.28%)	5.1593	0.0231	0.138	0.545 (0.322-0.922)	202 (55.95%)	180 (52.63%)	0.782	0.3765	0.8316	1.14 (0.850-1.54)
Single blind	3 (3.16%)	3 (2.13%)	#	0.687	0.8244	1.50 (0.269-7.59)	6 (1.66%)	5 (1.46%)	0.046	0.8308	0.8316	1.14 (0.344-3.77)
Double blind	23 (24.21%)	34 (24.11%)	0.0003	0.9864	0.986	1.01 (0.548-1.85)	50 (13.85%)	57 (16.66%)	1.079	0.2988	0.8316	0.804 (0.532-1.21)
Double-blind, double-dummy	10 (10.53%)	10 (7.09%)	0.863	0.3529	0.52 8	1.54 (0.615-3.86)	-	-	-	-	-	-
Triple blind	8 (8.42%)	5 (3.55%)	2.5915	0.1074	0.214	2.50 (0.793-7.89)	29 (8.03%)	26 (7.60%)	0.045	0.8316	0.8316	1.06 (0.612-1.84)
Quadruple	8 (8.42%)	4 (2.84%)	#	0.0713	0.213	3.15 (0.921-10.8)	74 (20.49%)	74 (21.63%)	0.137	0.7112	0.8316	0.934 (0.650-1.34)

For recruitment status, the total number of trials in the "not yet recruiting" phase in the pre-COVID period was 19 vs 48 in the post-COVID period. Corresponding numbers were 275 and 114 for "recruitment closed", 19 and 240 for "open to recruitment", 6 and 1 for "suspended", 40 and 58 for "active but not yet recruiting", 77 and 14 for "terminated", 7 and 8 for "withdrawn", and 13 and 0 for "unknown" for the pre-COVID and post-COVID periods, respectively. In India, there was a significant increase in trials in the "open to recruitment" phase (p-value = 0.00025) and trials in the "not yet recruiting" phase (p-value = 0.02017). There was also a significant decline in trials in the "recruitment closed" phase (p-value = 0.00025). A declining trend was seen in the number of terminated trials in India after COVID; however, the change was found to be non-significant (p-value = 0.05025).

In the USA, there was a significant increase in trials in the "open to recruitment" phase and a significant decline in trials in the "recruitment closed" phase (p-value = 0.0002667 for both). The USA also showed a significant increase in trials in the "active but not yet recruiting" phase (p-value = 0.0392). There was a significant decline in trials with recruitment status "terminated" (p-value = 0.0002667) and "unknown" (p-value = 0.0004) (Table 4).

**Table 4:** Distribution of trials as per recruitment status Data are presented as numbers and percentages. Categorical data were analysed using Pearson's chi-square test or Fisher's exact test, as appropriate. # indicates that Fisher's exact test was applied; therefore, no χ² statistic is reported. Adjusted p-values were obtained using the Benjamini-Hochberg procedure to control the false discovery rate. Odds ratios (ORs) are presented with 95% confidence intervals (CIs) and represent the odds of a trial belonging to the given category in the pre-COVID period relative to the post-COVID period. * represents an adjusted p-value < 0.05.

Recruitment status	India						USA					
	Pre-COVID period n=95	Post-COVID period n=141	χ²	P-value	Adjusted P-value	OR (95% CI)	Pre-COVID period n=361	Post-COVID period n=342	χ²	P-value	Adjusted P-value	OR (95% CI)
Not yet recruiting	17 (17.89%)	46 (32.62%)	6.2927	0.0121	0.02017*	0.450 (0.239-0.847)	2 (0.55%)	2 (0.58%)	#	1	1	0.947 (0.133-6.76)
Recruitment closed	60 (63.16%)	44 (31.21%)	23.5098	0.0001	0.00025*	3.78 (2.18-6.54)	215 (59.55%)	70 (20.46%)	111.321	0.0001	0.0002667*	5.72 (4.09-8.01)
Open to recruitment	10 (10.53%)	50 (35.46%)	18.6122	0.0001	0.00025*	0.214 (0.102-0.449)	9 (2.49%)	190 (55.55%)	243.658	0.0001	0.0002667*	0.0205 (0.0102-0.0410)
Suspended	3 (3.15%)	0 (0%)	#	0.064	0.064	10.7 (0.547-210)	3 (0.83%)	1 (0.29%)	#	0.6245	0.8156571	2.86 (0.296-27.6)
Active but not yet recruiting	-	-	-	-	-	-	40 (11.08%)	58 (16.95%)	5.059	0.0245	0.0392*	0.610 (0.396-0.941)
Terminated	5 (5.26%)	1 (0.70%)	#	0.0402	0.05025	7.78 (0.894-67.7)	72 (19.94%)	13 (3.80%)	43.06	0.0001	0.0002667*	6.31 (3.42-11.6)
Withdrawn	-	-	-	-	-	-	7 (1.93%)	8 (2.33%)	0.135	0.7137	0.8156571	0.826 (0.296-2.30)
Unknown	-	-	-	-	-	-	13 (3.60%)	0 (0%)	#	0.0002	0.0004*	26.5 (1.57-448)

The total number of trials with national study sites was 221 in the pre-COVID period and 226 in the post-COVID period, whereas studies with multinational sites were 235 and 257 in the pre- and post-COVID periods, respectively. In India, the proportion of trials with study sites being national vs multinational remained constant across both periods, with about two-thirds being national and one-third multinational. Conversely, in the USA, the percentage of trials with study sites being national decreased from 44% (n = 159) in the pre-COVID period to 39.50% (n = 135) in the post-COVID period, whereas trials with multinational sites increased from 56% (n = 202) in the pre-COVID period to 60.50% (n = 207) in the post-COVID period; however, these changes were not found to be statistically significant.

In India, the median number of sites per study decreased in the post-COVID period, and on analysis by the Mann-Whitney U test, the change was found to be statistically significant (p-value = 0.0335). The USA showed contrasting findings with an increase in the median number of sites per study; however, the change was not found to be statistically significant (p-value = 0.0863). The median sample size per study decreased in India, whereas it increased in the USA; however, the changes in sample size in both countries were not found to be statistically significant (Table 5).

**Table 5:** Comparison of the number of study sites and sample size per study in India and the USA during pre- and post-COVID periods Data are presented as median (interquartile range). Quantitative data were analysed using the Mann-Whitney U test. Effect size is reported as rank-biserial correlation coefficient. * represents p value <0.05. IQR: interquartile range.

Variables	India					USA				
	Pre-COVID period n=95 Median (IQR)	Post-COVID period n=141 Median (IQR)	U statistic	P-value	Rank-biserial correlation coefficient	Pre-COVID period n=361 Median (IQR)	Post-COVID period n=342 Median (IQR)	U statistic	P-value	Rank- biserial correlation coefficient
Number of sites per study	13 (7-21)	10 (6-15.5)	5605	0.0335*	-0.163	18 (4-71.5)	23 (6-86)	57120.5	0.0863	0.0747
Sample size per study	168 (56-252)	120 (49-228)	5863	0.1048	-0.125	132 (60-393)	150 (72-402)	59474.5	0.4019	0.0366

## Discussion

In this study, we observed an overall increase in the number of regulatory clinical trials for NCDs in the post-COVID period, with contrasting trends between India and the USA, where the number of trials increased in India but declined slightly in the USA. The overall distribution of trial phases, study designs, and comparator use remained largely similar in both periods. There were changes seen in terms of therapeutic area. That is, in India, trials for diabetes showed a significant decline, while COPD showed a significant increase. Meanwhile, in the USA, trials for hypertension showed an increasing trend; however, this change was not statistically significant. Changes were also seen in recruitment status and number of sites, reflecting evolving patterns in clinical trial activity in the post-pandemic period.

Non-communicable diseases are a leading cause of morbidity and mortality, accounting for 75% of all non-pandemic-related deaths. In 2021, NCDs caused 43 million deaths, out of which 18 million deaths occurred before the age of 70 years, and a large majority of these premature deaths were in low- and middle-income countries. Cardiovascular diseases account for most NCD deaths (19 million deaths in 2021), followed by cancers (10 million), chronic respiratory diseases (4 million), and diabetes (over 2 million). Collectively, these four groups of diseases account for 80% of all premature NCD deaths [7]. As per Indian data, NCDs are estimated to account for 63% of all deaths in the country, of which cardiovascular diseases lead with 27% overall mortality cause, followed by chronic respiratory diseases (11%), cancers (9%), diabetes (3%), and others (13%) [10]. Cancer accounted for an estimated 19.3 million new cases and almost 10.0 million deaths in 2020 worldwide [11]. Breast cancer is the most common cause of cancer in the USA as well as in India [8,9]. Based on their high global and national burden, these conditions were selected in the present study.

The COVID-19 pandemic placed a major burden on healthcare systems worldwide and significantly affected clinical research activities. Globally, the number of newly enrolled patients in clinical trials decreased by 65% in March 2020 and by 79% in April 2020 compared to the corresponding months in 2019. In India, the decline was even greater, with new patient enrolment decreasing by 84% in March 2020 and 97% in April 2020 compared to the previous year. The therapeutic areas affected the most include endocrine (80%), cardiovascular (69%), CNS (68%), oncology (48%), and respiratory (33%) [2]. The major challenges that interfered with the routine conduct of clinical research included restricted access to study sites due to nationwide lockdowns and suspension of non-emergency services, participant hesitation affecting subject enrolment and recruitment, as well as higher dropout rates, increased risk of COVID-19 exposure to participants and trial staff, operational challenges such as delivering investigational products and study medications and providing personal protective equipment, obstacles in on-site monitoring and audits, disruptions in trial timelines due to delays in assessments and follow-up visits, and emergency changes in trial protocols [2]. Overall, these challenges reflect the widespread impact of the COVID-19 pandemic on clinical research. To evaluate whether clinical research has recovered from this impact or whether the pandemic has left long-term consequences, we compared the clinical trial data for NCDs from 24 months (two years) before the start of the pandemic to 24 months after the end of the pandemic.

Our findings revealed different trends in India and the USA. India showed an increase in the total number of registered trials from 95 to 141. In contrast, the USA demonstrated a slight decline from 361 to 342 trials. The increase in trial activity in India may reflect its increasing recognition as an important destination for clinical research [12]. A diverse racial and ethnic population, large pool of treatment-naïve patients, lower costs, qualified medical professionals, widespread use of English in the health sector, numerous urban centres with high standards of care and cutting-edge health infrastructure, and good alignment of Indian regulatory guidelines with FDA and European Medicines Agency (EMA) guidelines make India an attractive destination for multinational clinical trials [13]. Additionally, the recovery of healthcare systems after the pandemic may have facilitated the resumption of paused trials along with the initiation of new studies, leading to an overall increase in trial registrations. The modest decline in overall USA trials for NCDs and the significant decline in trials for diabetes mellitus in India might reflect a shift in research priorities toward communicable diseases and COVID-19 [14].

Regarding therapeutic areas, the increasing trend seen in trials for COPD and asthma in both countries might be explained by an increased focus on pulmonary research following the pandemic [15]. In the USA, trials for hypertension showed a rising trend. A possible explanation for this might be an increased interest in hypertension research, as it was the most common comorbidity associated with severe COVID outcomes and the increased incidence of new-onset hypertension post COVID [16,17]. However, this finding was not statistically significant, and therefore this explanation should be considered as a possible hypothesis.

Phase 4 trials primarily focus on post-marketing surveillance and long-term safety. The significant decline in Phase 4 trials in the USA could be associated with resource optimization, where Phase 4 trials took a relative backseat compared to other research activities; however, there could be other contributory factors as well.

The decrease in the number of study sites in India in the post-COVID period may be explained by clinical trial operations not yet returning to full-fledged scale, with a more gradual expansion of sites instead of large multicentric studies, as a result of caution on the part of sponsors. Additionally, this change could be explained by adoption of alternative approaches, such as remote monitoring and elements of decentralized clinical trials [18]. However, the underlying reasons were not directly assessed.

The authors hypothesize that the significant increase in the number of trials with ongoing recruitment, along with a significant decrease in trials with recruitment closed, may be attributed to a possible backlog effect, where trials that were probably paused during the pandemic are now resumed, and new trials initiated more recently are still in their early stages and therefore have had less time to complete recruitment. Regarding the number of trials terminated, there was a statistically significant decline in the post-COVID period in the USA and a declining trend observed in India, which may be due to a large number of trials initiated in the pre-COVID phase that possibly got terminated after the onset of the pandemic.

Despite these operational changes, fundamental trial designs remained constant. Randomised parallel designs were predominant, and the distribution of comparator types remained stable across both periods. This suggests that the core methodological framework of clinical trials was largely preserved.

The strengths of this study include the use of established clinical trial registries for data collection. The limitations include heavy reliance on the accuracy of registry data and updates, which are dynamic and may not be updated regularly or filled in accurately. As the objective of the study was to compare clinical trial activity during the pre-pandemic and post-pandemic periods, the intervening pandemic period was not included in the analysis, and therefore no information can be provided about the disruption of trial activity during the pandemic itself. As the objective was to compare regulatory trial activity within India and the USA, other international and national registries were excluded, which may introduce selection bias and limit the generalisability of the findings. In addition, the study did not directly assess changes in regulatory policies, pharmaceutical investment trends, decentralized clinical trial adoption, or other factors that may have influenced trial activity during the study period; therefore, changes observed in this study cannot be attributed to the pandemic alone. Observational studies, device studies, and non-regulatory trials were not included; therefore, the findings do not represent the entire spectrum of clinical research activity in NCDs.

Our findings suggest that, while drug clinical trial activity has largely recovered, there are some adaptations in the post-pandemic period. Future research should be directed towards qualitative studies to explore the underlying reasons for these changes by investigating the perspectives of various industry stakeholders to better understand and confirm the factors influencing the conduct of clinical trials in the post-pandemic era.

## Conclusions

The adverse impact of the COVID-19 pandemic on clinical research has been largely overcome, and the number of trials has almost returned to pre-pandemic levels. However, there has been some impact on certain elements of regulatory clinical trials for NCDs. India showed an overall increase in the number of registered trials, while the USA showed a slight decline. In India, there was a significant rise in COPD trials, while trials for diabetes declined significantly. Phase 4 trials in the USA demonstrated a statistically significant decline. There was a significant decrease in trials with recruitment closed in both India and the USA, whereas trials that were still open to recruitment showed a significant increase in both countries. In India, post-COVID, a significantly greater number of trials were in the not yet recruiting phase, while in the USA, there was a significant rise seen in active but not yet recruiting trials and a significant decline seen in terminated trials and trials with an unknown recruitment status. The median number of study sites was significantly reduced in the post-COVID period in India.

## Figures and Tables

**Figure 1:**
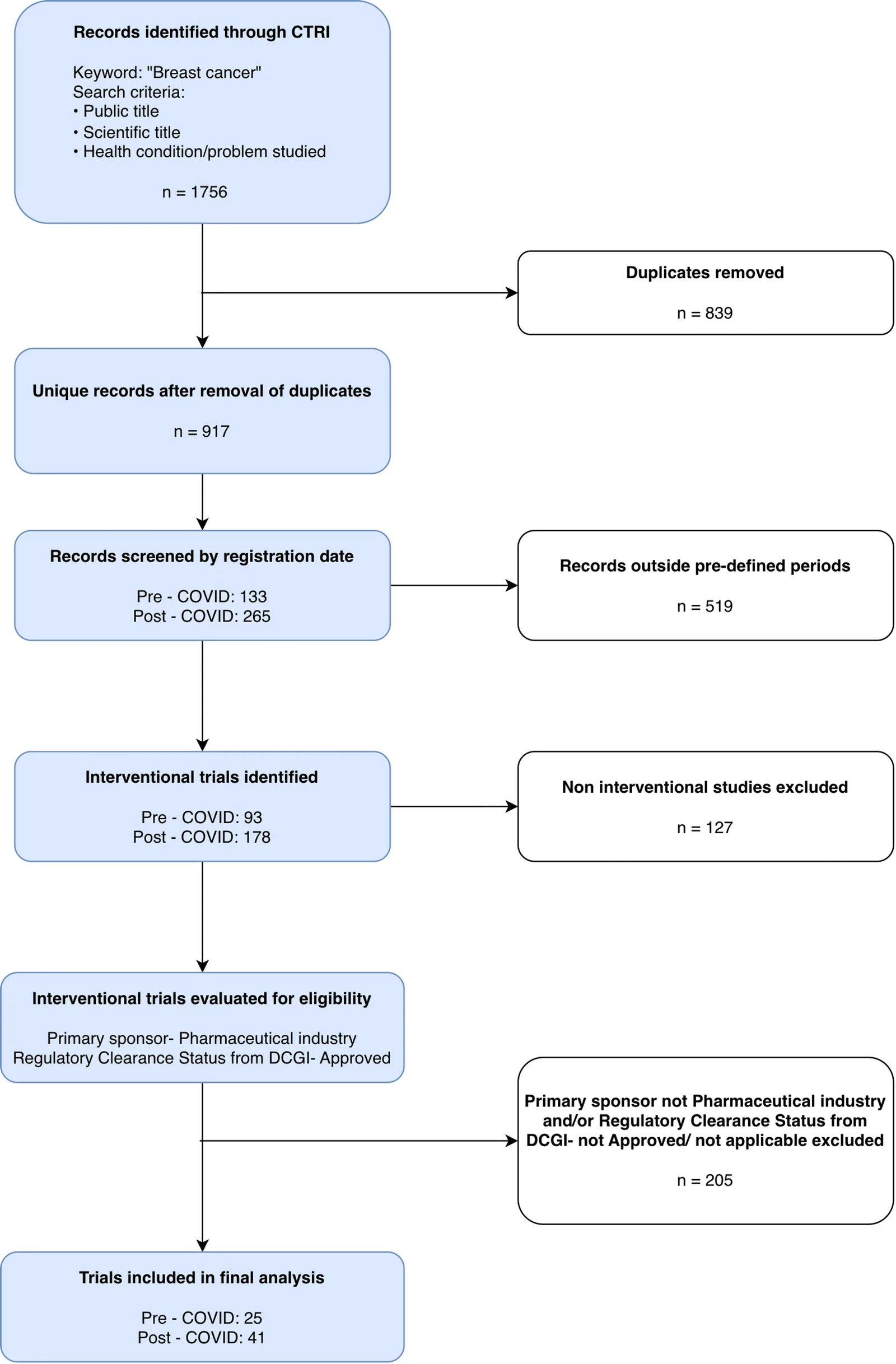
Search strategy and study selection flow diagram for breast cancer clinical trials in the Clinical Trials Registry - India (CTRI)

**Figure 2:**
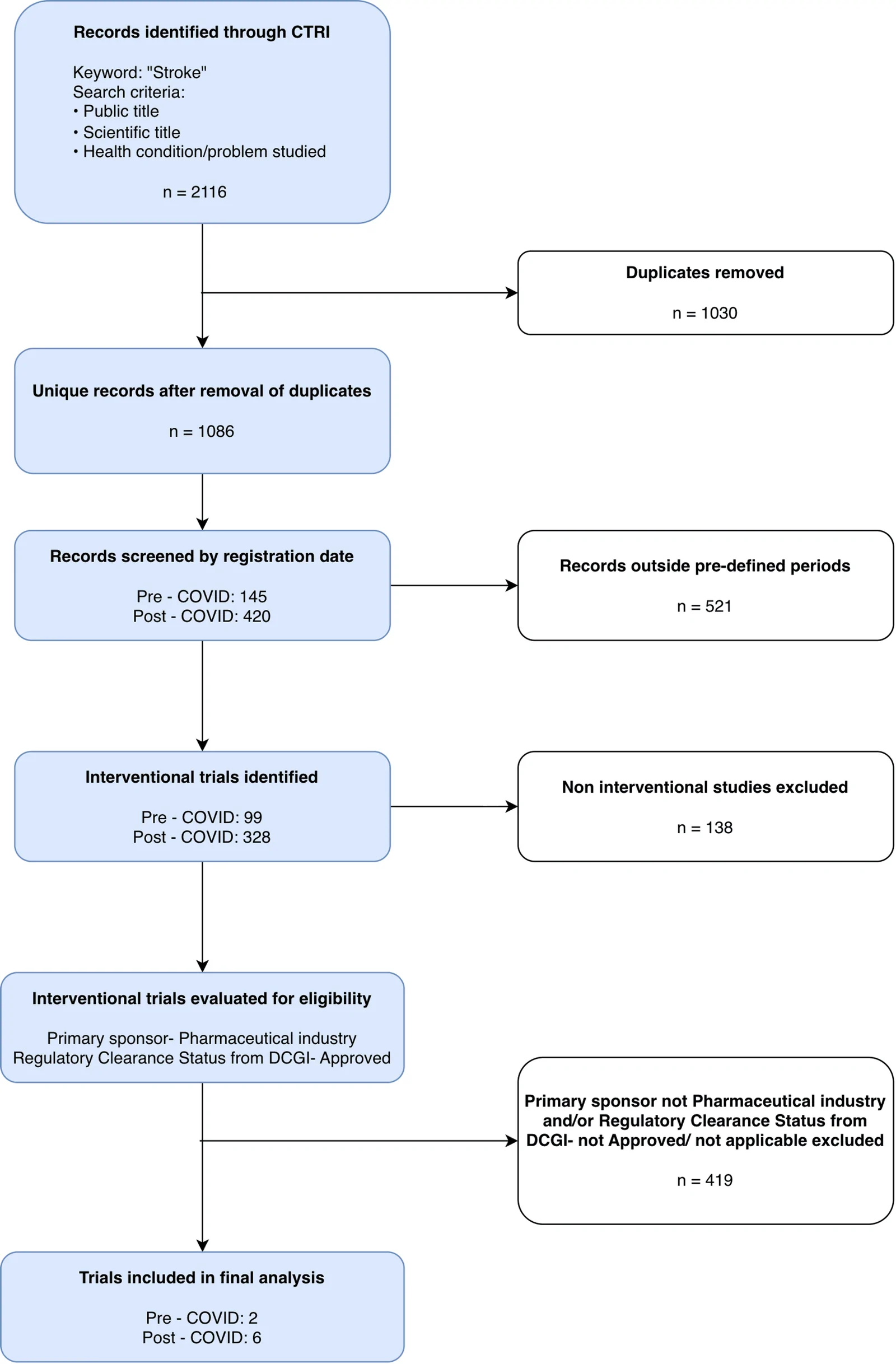
Search strategy and study selection flow diagram for stroke clinical trials in the Clinical Trials Registry - India (CTRI)

**Figure 3:**
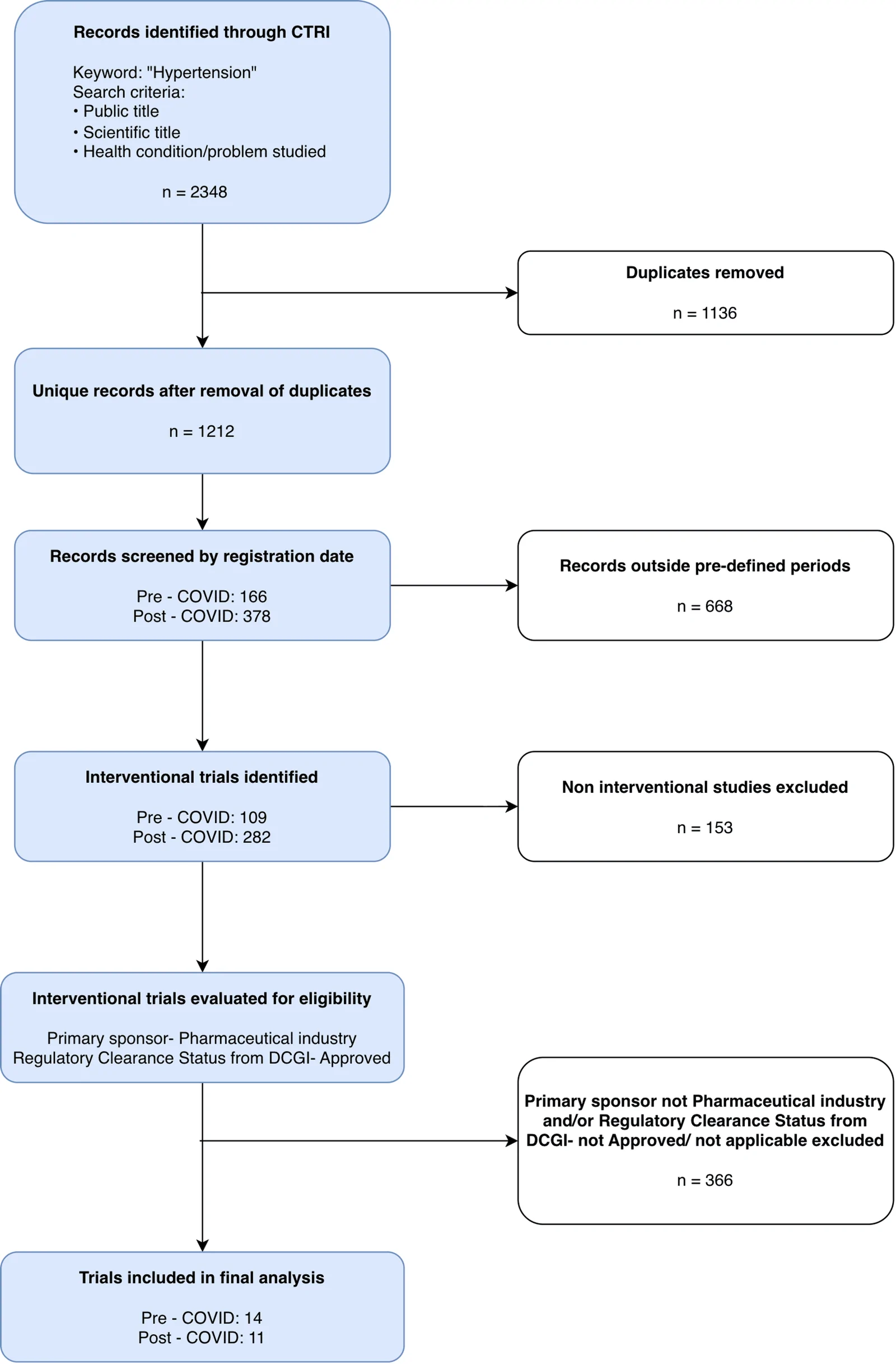
Search strategy and study selection flow diagram for hypertension clinical trials in the Clinical Trials Registry - India (CTRI)

**Figure 4:**
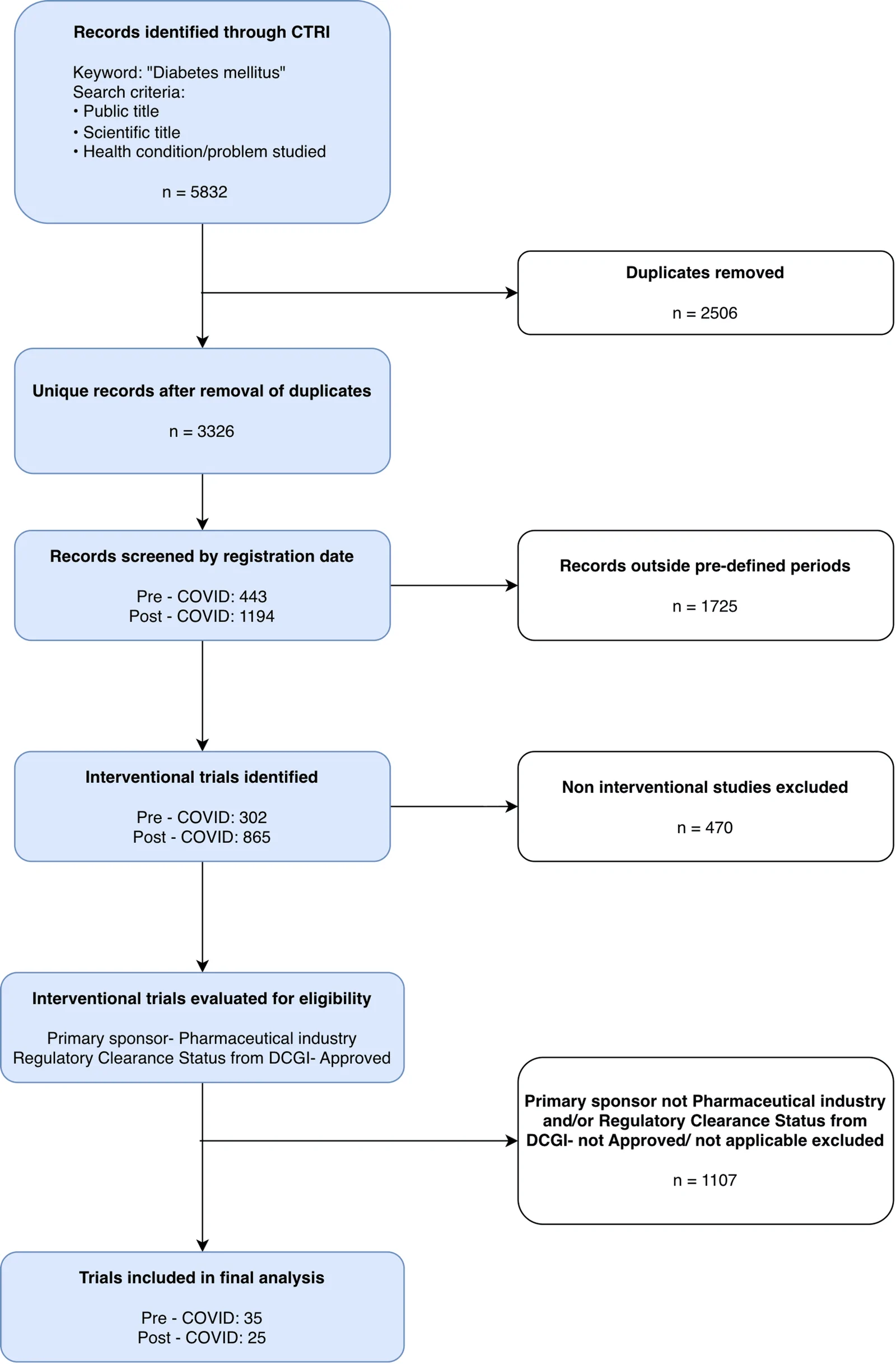
Search strategy and study selection flow diagram for diabetes mellitus clinical trials in the Clinical Trials Registry - India (CTRI)

**Figure 5:**
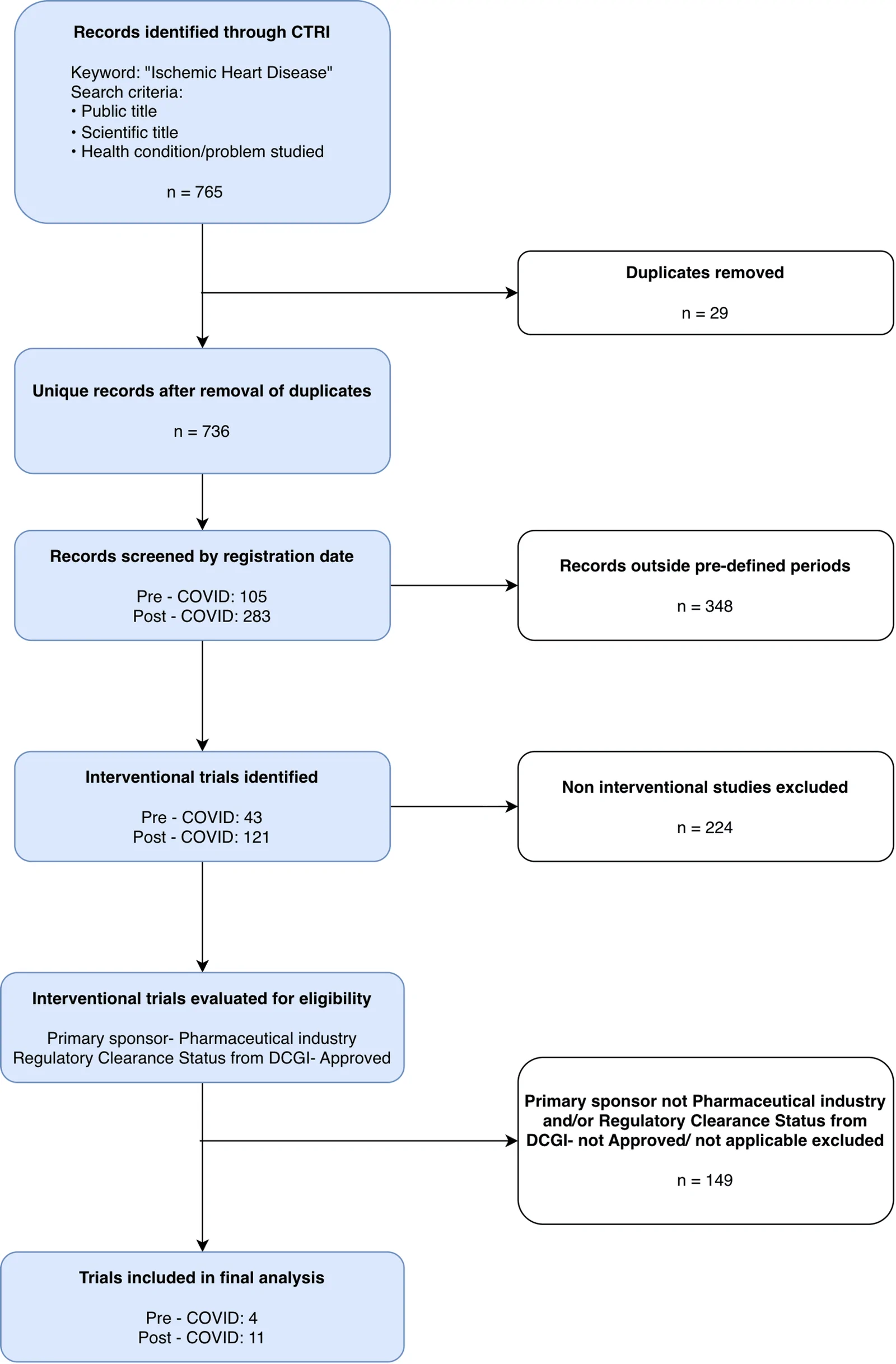
Search strategy and study selection flow diagram for ischemic heart disease clinical trials in the Clinical Trials Registry - India (CTRI)

**Figure 6:**
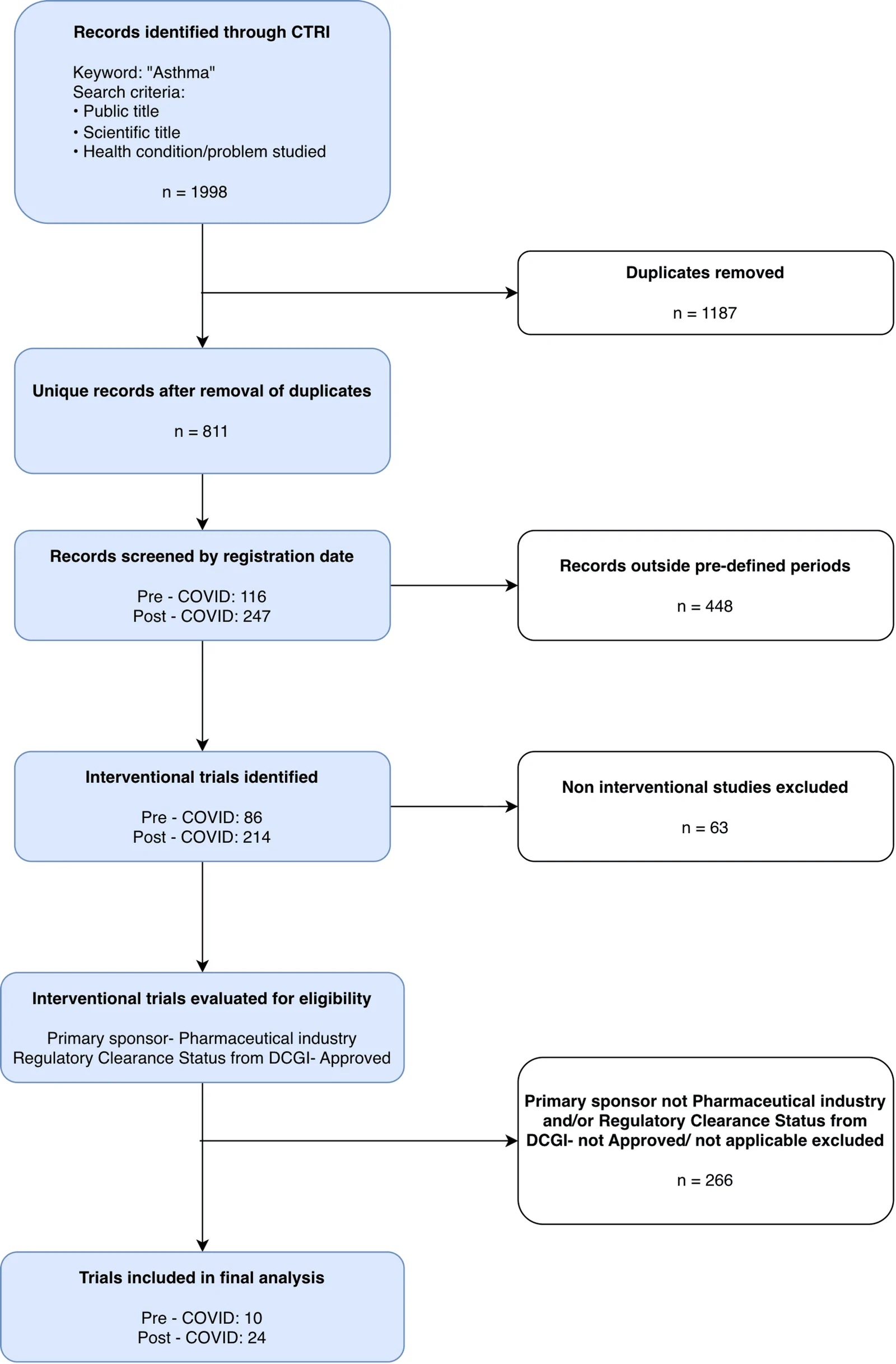
Search strategy and study selection flow diagram for asthma clinical trials in the Clinical Trials Registry - India (CTRI)

**Figure 7:**
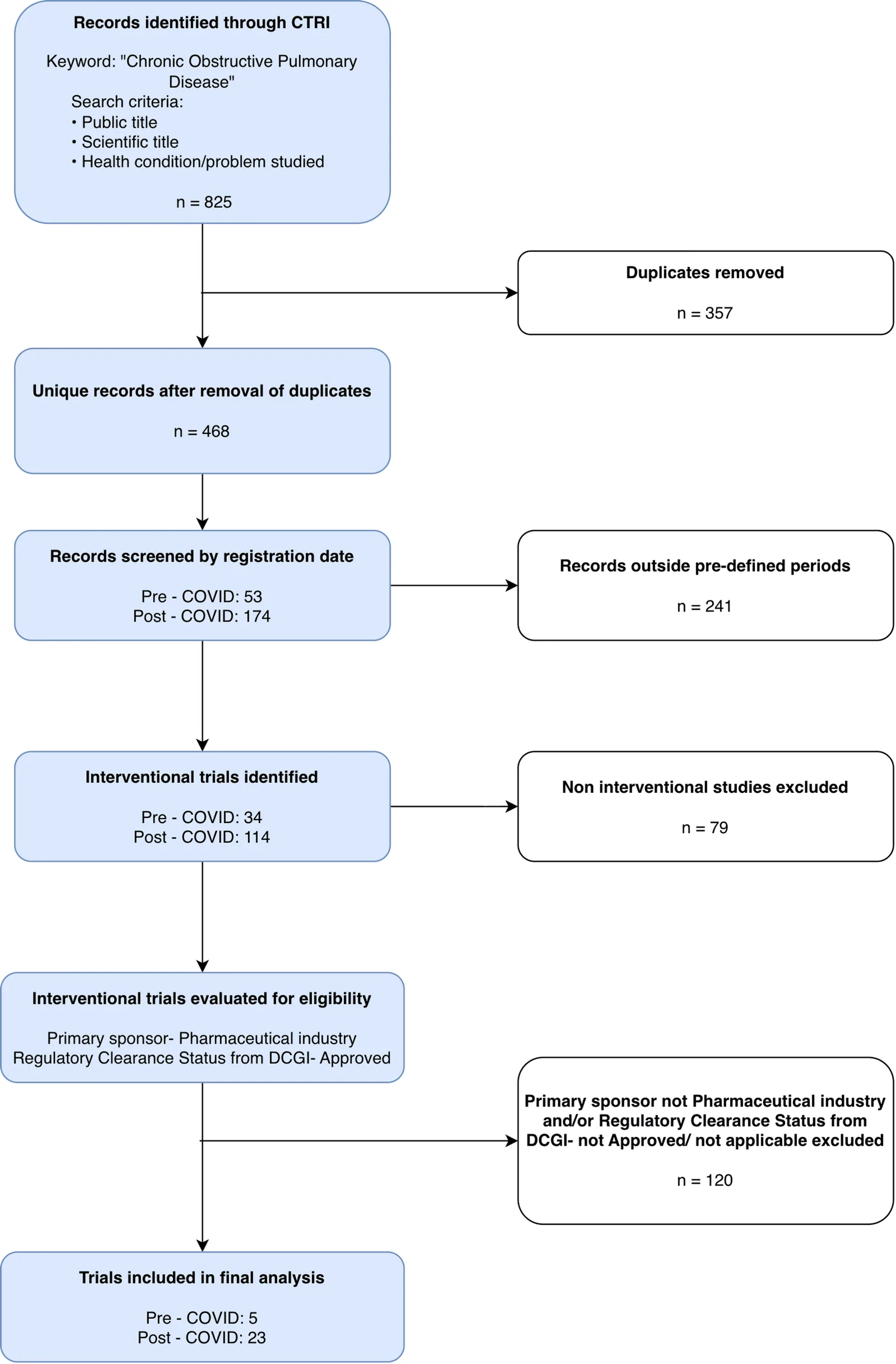
Search strategy and study selection flow diagram for chronic obstructive pulmonary disease (COPD) clinical trials in the Clinical Trials Registry - India (CTRI)

**Figure 8:**
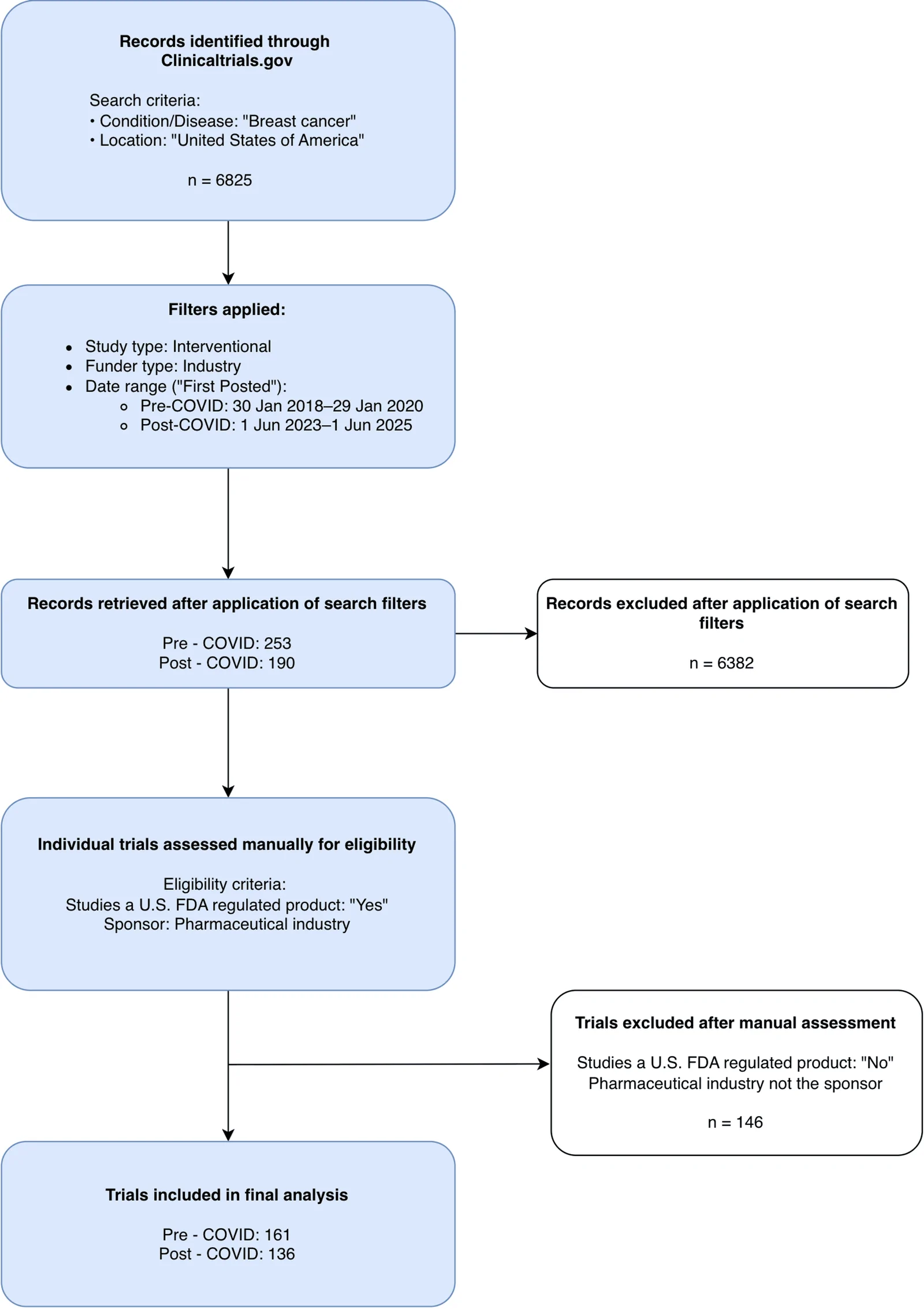
Search strategy and study selection flow diagram for breast cancer clinical trials on the ClinicalTrials.gov website

**Figure 9:**
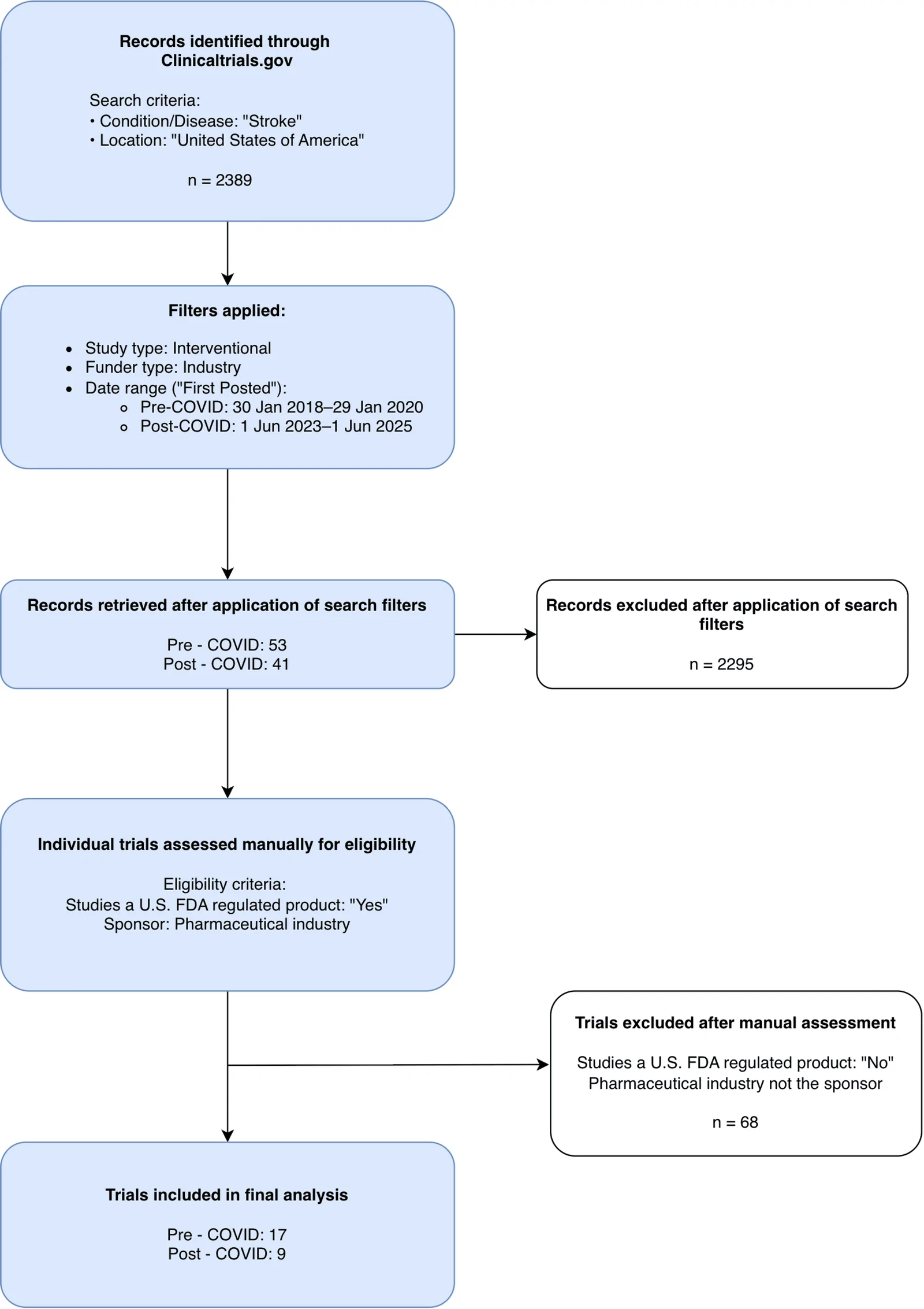
Search strategy and study selection flow diagram for stroke clinical trials on the ClinicalTrials.gov website

**Figure 10:**
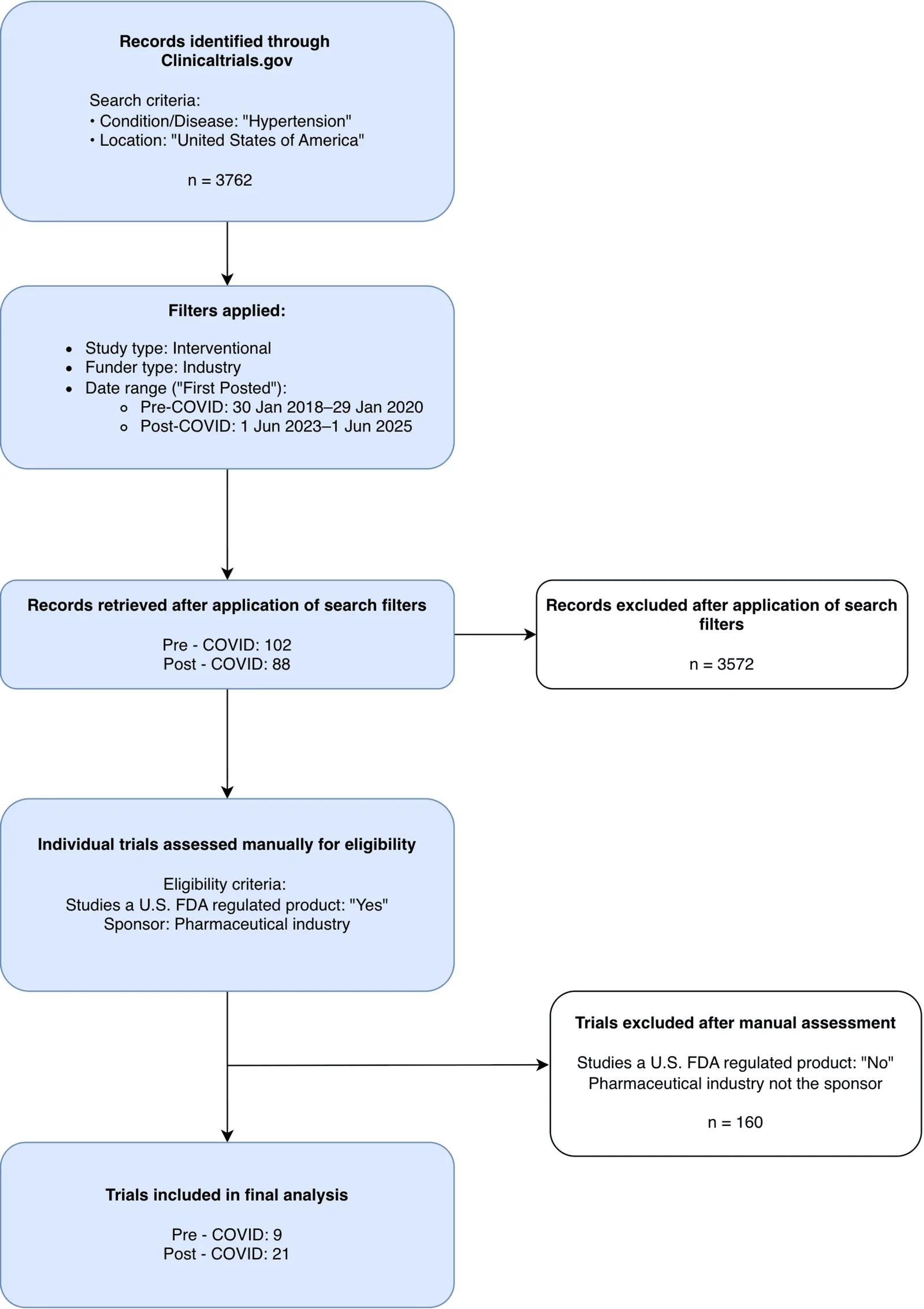
Search strategy and study selection flow diagram for hypertension clinical trials on the ClinicalTrials.gov website

**Figure 11:**
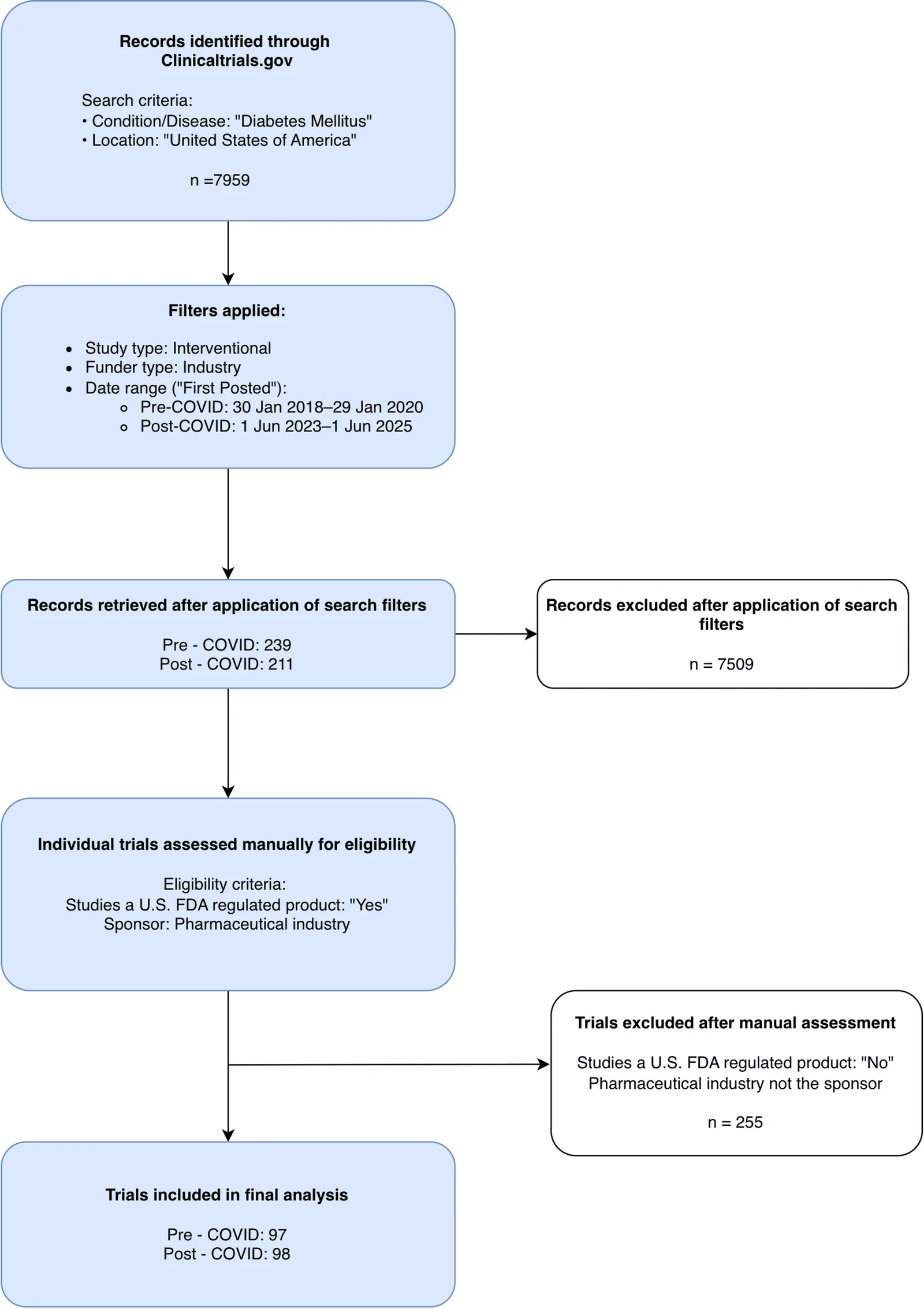
Search strategy and study selection flow diagram for diabetes mellitus clinical trials on the ClinicalTrials.gov website

**Figure 12:**
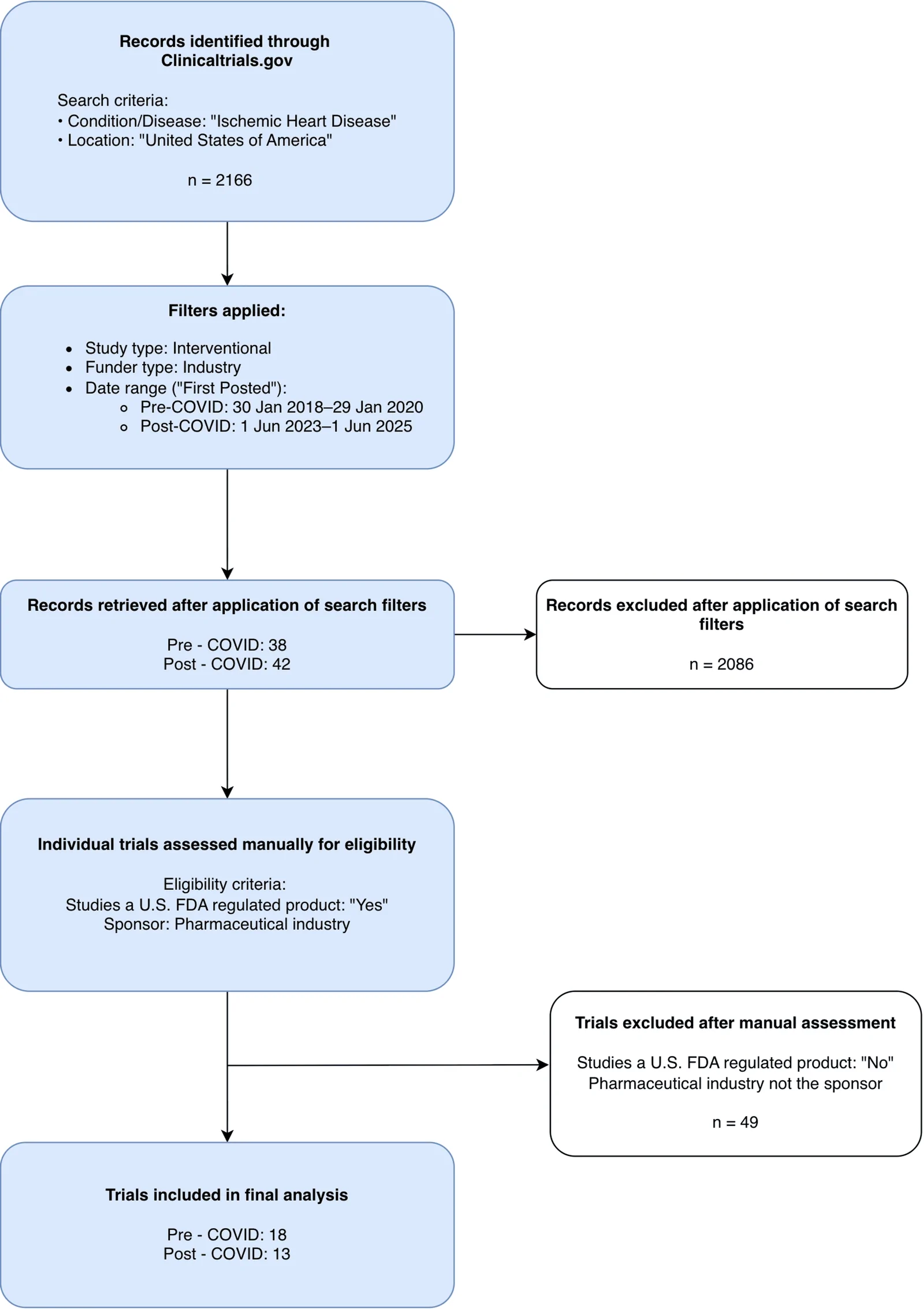
Search strategy and study selection flow diagram for ischemic heart disease clinical trials on the ClinicalTrials.gov website

**Figure 13:**
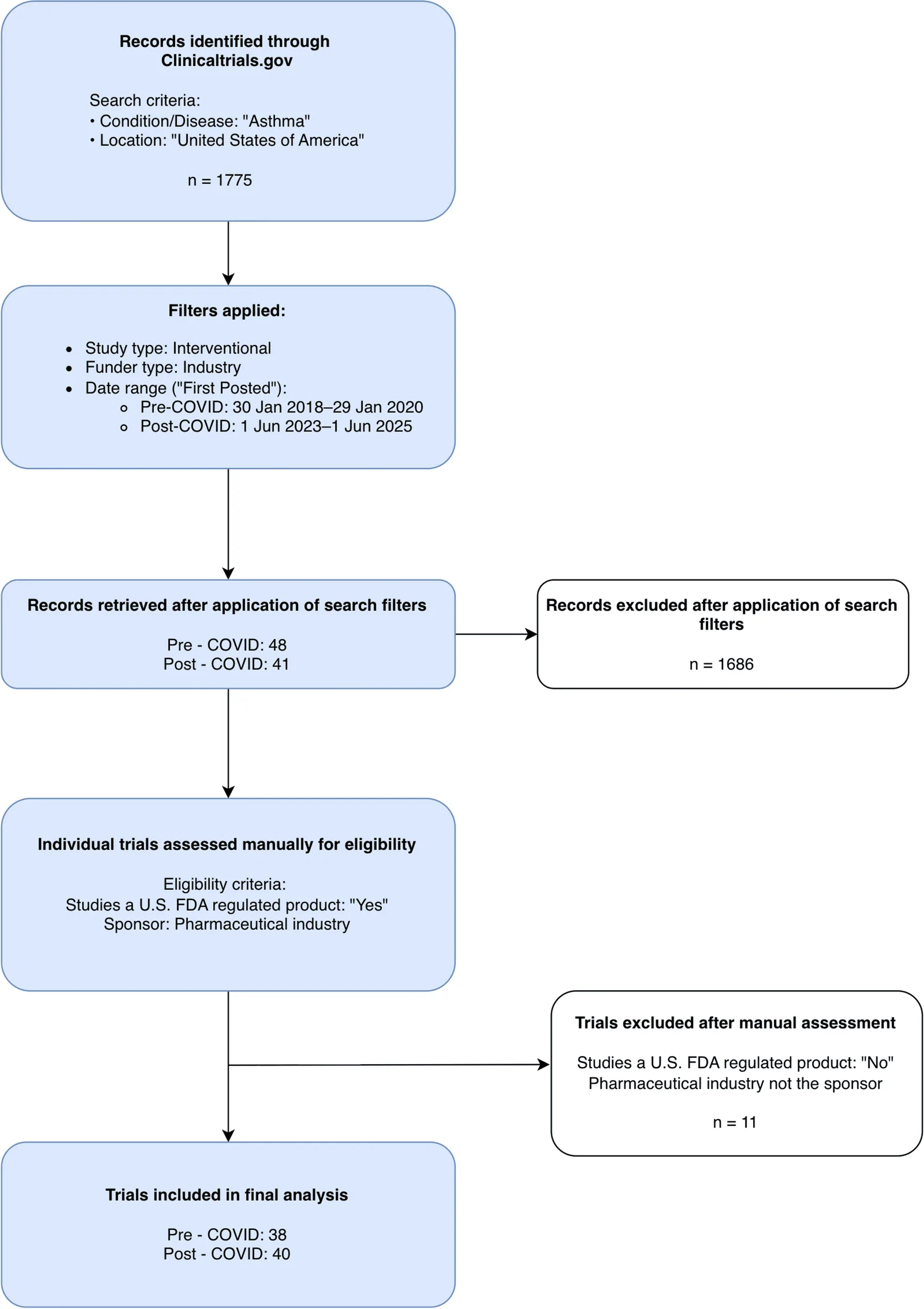
Search strategy and study selection flow diagram for asthma clinical trials on the ClinicalTrials.gov website

**Figure 14:**
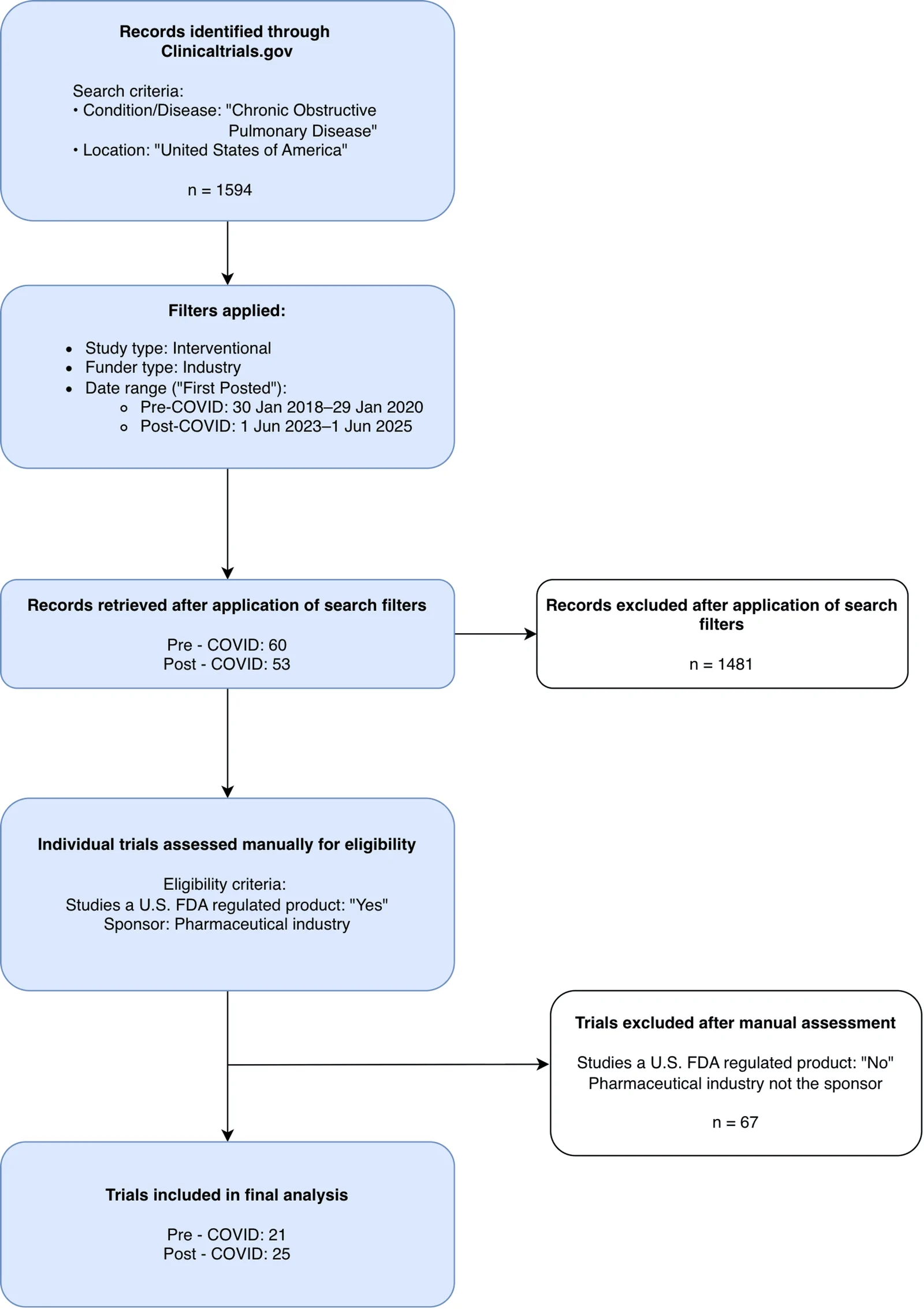
Search strategy and study selection flow diagram for chronic obstructive pulmonary disease (COPD) clinical trials on the ClinicalTrials.gov website

## Appendices

Search strategy and study selection for India

Clinical trial records for each of the seven selected non-communicable diseases were searched separately on the Clinical Trials Registry - India (CTRI) website using the "Advanced Search" function. In the "Keyword" Search bar, the respective disease name was entered as the search term. Under "Choose Search Strategy", the keyword was searched separately using the following three fields: "Public Title of Study", "Scientific Title of Study", and "Health Condition/Problem Studied".

Records retrieved using the three search strategies were reviewed independently by two reviewers, and overlapping records were removed as duplicates. The remaining records after removal of duplicates were screened according to their registration dates, and trials falling within the predefined pre-COVID-19 period (30 January 2018 to 29 January 2020) and post-COVID-19 period (1 June 2023 to 1 June 2025) were identified. Records registered outside these periods were excluded.

Among the trials identified within the predefined study periods, interventional trials were retained, while non-interventional studies were excluded. The interventional trials were then manually screened by reviewing the individual trial records for "primary sponsor" and "regulatory clearance status from DCGI". Trials with "pharmaceutical industry" as the primary sponsor and DCGI regulatory clearance status marked as "Approved" were considered eligible and included in the final analysis. All included trial records contained complete information for the variables analysed and therefore no record was excluded on account of missing data. Any discrepancies between the reviewers were resolved through discussion. Data from the retrieved records were entered into a standardized case record form prepared in Microsoft Excel.

The registry search for each disease was performed at different time points during the data-collection period. The corresponding search terms and search dates for each disease are provided in Table 6. The disease-specific screening and selection process is presented in Figures 1-7.

**Table 6:** Registry search details for Indian clinical trials

Disease	Search term	Search period
Breast cancer	breast cancer	July 2025
Stroke	stroke	August 2025
Hypertension	hypertension	July 2025
Diabetes mellitus	diabetes mellitus	June 2025
Ischemic heart disease	ischemic heart disease	June 2025
Asthma	asthma	August 2025
Chronic obstructive pulmonary disease (COPD)	chronic obstructive pulmonary disease	September 2025

Search strategy and study selection for the USA

A search was conducted on ClinicalTrials.gov for the seven non-communicable diseases included in the study, namely, diabetes mellitus, breast cancer, stroke, hypertension, ischemic heart disease, asthma, and chronic obstructive pulmonary disease. The same disease-specific search terms used for the India search were entered in the "Condition/Disease" search field, with "United States of America" selected under the Location field. The search was conducted separately for each disease. The following filters were then applied: Study Type - "Interventional", Funder Type - "Industry", and in the Date range under "First Posted" the predefined pre-COVID (30 January 2018 to 29 January 2020) and post-COVID (1 June 2023 to 1 June 2025) periods were entered. All records retrieved after application of these filters were manually assessed for eligibility. Studies were excluded if "Studies a U.S. FDA-Regulated Drug Product" was marked "No" or if the sponsor was not a pharmaceutical company. Records retrieved using the search strategy were reviewed independently by two reviewers. Any discrepancies between the reviewers were resolved through discussion. Data from the retrieved records were entered into a standardized case record form prepared in Microsoft Excel. All included trial records contained complete information for the variables analysed, and therefore no record was excluded on account of missing data.

The registry search for each disease was performed at different time points during the data-collection period. The corresponding search terms and search dates for each disease are provided in Table 7. The disease-specific screening and selection process is presented in Figures 8-14.

**Table 7:** Registry search details for USA clinical trials

Disease	Search term	Search period
Breast cancer	breast cancer	November 2025
Stroke	stroke	December 2025
Hypertension	hypertension	January 2026
Diabetes mellitus	diabetes mellitus	October 2025
Ischemic heart disease	ischemic heart disease	January 2026
Asthma	asthma	February 2026
Chronic obstructive pulmonary disease	chronic obstructive pulmonary disease	February 2026
